# Risk factors of hematoma after SWL for renal calculi: analysis from RCTs and a literature review

**DOI:** 10.1007/s11255-024-04205-3

**Published:** 2024-09-18

**Authors:** Chris Ho-Ming Wong, Ivan Ching-Ho Ko, Emmy Sui-Fan Tang, Steffi Kar-Kei Yuen, David Ka-Wai Leung, Angel Wing-Yan Kong, Peter Ka-Fung Chiu, Jeremy Yuen-Chun Teoh, Chi Fai Ng

**Affiliations:** 1 Department of Surgery, SH Ho Urology Centre, The Chinese University of Hong Kong, Clinical Sciences Building, Prince of Wales, Hospital, New Territories, Hong Kong SAR; 2 Department of Urology, Medical University of Vienna, Vienna, Austria; 3 Li Ka Shing Institute of Health Sciences, The Chinese University of Hong Kong, Hong Kong, China

**Keywords:** Urolithiasis, Extracorporeal shockwave lithotripsy, Perinephric hematoma, Predicting factors, Complications, Stepwise ramping protocol

## Abstract

**Objective:**

To identify risk factors of perinephric hematoma following extracorporeal shockwave lithotripsy (SWL) for renal calculi through combined analysis of two randomized controlled trials.

**Patients and methods:**

This post-hoc analysis included adult patients with solitary renal calculi ranging from 5 to 15 mm, treated with SWL between 2016 and 2022. All patients received cross-sectional imaging (either non-contrast computer tomography scan or magnetic resonance imaging) two days post-SWL to assess the presence and severity of perinephric hematoma.

**Results:**

Among 573 patients analyzed, 173 (30.9%) developed perinephric hematoma by Day 2 post-SWL. Multivariate logistic regression identified higher total energy delivered (odds ratio [OR] = 1.533, *p* = 0.003), higher mean stone density (OR = 2.603, *p* = 0.01), higher maximal stone density (OR = 3.578, *p* = 0.03), and lower pole stone location (OR = 1.545, *p* = 0.029) were risk factors for the development of hematoma. Conversely, the stepwise ramping protocol was a protective factor for hematoma formation. (OR = 0.572, *p* = 0.042).

**Conclusions:**

This study elucidates key factors influencing the risk of perinephric hematoma post-SWL, highlighting the importance of procedural adjustments such as the stepwise ramping protocol to reduce complications. These insights call for targeted patient and treatment strategy optimization to enhance SWL safety and efficacy.

## Introduction

Extracorporeal shockwave lithotripsy (SWL) has revolutionized the treatment of renal calculi since its introduction in 1980 [1]. This non-invasive technique has become a widely accepted first-line therapy for many patients with kidney stones due to its effectiveness and favorable safety profile [2, 3]. However, despite its advantages, SWL is not without complications. One of the most serious and potentially life-threatening complications is the development of perinephric hematoma.

The reported incidence of perinephric hematoma following SWL varies considerably in the literature, largely depending on the study design and imaging modality used. The rates were reported from below 1% to up to 30%. Numerous risk factors have been suggested to contribute to the development of perinephric hematoma after SWL. These factors can be broadly categorized into patient-related, stone-related, and treatment-related factors. Patient-related factors include advanced age, hypertension, and the use of anticoagulant or antiplatelet medications. Stone characteristics, such as size, location, and density, have also been implicated. Furthermore, treatment-related factors, such as the total energy, may influence the risk of hematoma formation [4].

Despite the identification of these potential risk factors, there is still a lack of consensus regarding their relative importance and the optimal strategies for minimizing the risk of perinephric hematoma. This can be attributed to the limited number of large, prospective studies and the heterogeneity of study designs and populations.

The current study aims to identify the risk factors of perinephric hematoma after SWL for renal calculi through a well-characterized prospective cohort with a standardized imaging protocol. We also aim to describe a literature review examining the existing studies on perinephric hematoma following SWL. The review will discuss the current state of knowledge regarding the incidence and risk factors of this complication. By synthesizing the available evidence, we aim to provide a broader context for our findings and highlight areas that warrant further research.

## Materials and methods

### Cohort design and patient population

This study involves a post-hoc analysis of two randomized controlled cohorts (CUHK_CCT00462 and CUHK_CCRB00510) conducted from 2016 to 2022 [5, 6]. Both trials enrolled adult patients with solitary renal calculi ranging from 5 to 15 mm, scheduled for primary SWL.

#### Inclusion criteria

- Adults aged 18 years and older.
- Patients with solitary radio-opaque renal stones, 5–15 mm in diameter.

#### Exclusion criteria

- Multiple renal calculi within the same calyx.
- Anatomical abnormalities of the renal or ureteric system, such as medullary sponge kidney, caliceal diverticulum, horseshoe kidney, or ureteropelvic junction obstruction.
- Previous treatments other than SWL as the primary intervention.
- Presence of a ureteric stent or nephrostomy tube within 3 months prior to SWL.
- Known history of cystine stones.

### Intervention

Treatment was administered using a Storz Modulith SLX-F2 electromagnetic lithotriptor, delivering a maximum of 3,000 shocks at a rate of 1.5 Hz. The recommended maximum energy level was set at 14 kV (Level 7). A portion of the cohort underwent a stepwise ramping protocol, receiving 1,000 shocks sequentially at energy levels of 10 kV, 12 kV, and finally 14 kV. Others were treated with an initial 100 shock for lower voltage and then continued with fixed voltage protocol at 14 kV. The cohort featured two focal zone settings: a standard focal zone (28 × 6 mm, 150Mpa) and an extended focal zone (50 × 9 mm, 90Mpa).

Procedure parameters, including stone location, size, density, and patient data such as age and anti-platelet medication use, were meticulously documented. Patients on baseline antiplatelet medication were advised to discontinue the drug 7 days prior to treatment.

### Follow-up of cohort and measurement of outcomes

Patients underwent clinical and radiological evaluations two days post-SWL. Those lacking either a CT or MRI at this follow-up were excluded from the analysis. The primary outcome measured was the presence of perinephric hematoma on Day 2 imaging, specifically noting the maximal thickness observed.

### Statistical analysis

Data were processed using SPSS version 24.0. Demographic statistics were analyzed via the Student t-test or Mann–Whitney U test, while categorical variables were assessed using chi-square or Fisher exact tests. Relevant variables were included in a multivariate logistic regression model to identify independent risk factors of perinephric hematoma post-SWL. Significance was set at p < 0.05.

## Results

A total of 573 patients met the inclusion and exclusion criteria and were analyzed. The mean age of the cohort was 56.0 ± 11.2. Fifty-two patients (9.1%) were on baseline anti-platelet medication, which was stopped at least 5–7 days before treatment. Two hundred and twenty-five patients (44.5%) had hypertension at the time of intervention. The mean value of the maximum stone dimension was 8.7 ± 2.9 mm. The mean stone density was 661 ± 293 Hounsfield Units (HU), and the mean maximal stone density of the cohort was 974 ± 347 HU. A majority of the stones treated were lower pole stones (*N* = 333, 58.1%). One hundred and forty-nine cases (26.0%) received a stepwise ramping protocol. One hundred and thirty-eight patients (24.1%) had their stones treated with an extended focal zone setting. The mean total energy delivered across the procedures was 269.7 Storz Medical Lithotripsy Index (SMLI). Outcomes were assessed with Day 2 imaging after initial SWL. Treatment success with resultant stone fragments < 4 mm was achieved in 400 cases (69.9%). Two hundred and forty-one cases (37.4%) were confirmed radiologically stone-free. The details were documented in Table 1.

**Table 1:** Background characteristics and treatment outcomes of the cohort

	N	%/SD/IQR
Mean age, SD	56	11.2
Mean BMI (kg/m^2^), SD	25.1	4.0
Hypertension, %	255	44.5%
Antiplatelet use, %	52	9.1%
Mean maximum stone diameter (mm), SD	8.7	2.9
Mean stone volume (mm^3^), SD	256.1	276.4
Mean maximum stone density (HU), SD	973.7	346.7
Mean stone density (HU), SD	661.6	292.8
Mean skin to stone distance (mm), SD	85.6	53.9
Mean cortical thickness (mm), SD	23.9	17.0
Stone laterality, %		
Left	330	57.6%
Right	243	42.4%
Stone site, %		
Renal pelvis	35	6.1%
Upper pole	80	14.0%
Middle pole	125	21.8%
Lower pole	333	58.1%
Stepwise ramping protocol	149	26.0%
Extended focal zone	138	24.1%
Mean total energy delivered (SMLI), SD	269.7	71.7
Treatment success, %	400	69.9%
Radiological stone free, %	214	37.4%
Development of perinephric hematoma, %	173	30.9%
Mean thickness of hematoma (mm), SD	8.4	1.7

*SD* standard deviation, *BMI* body mass index, *HU* Hounsfield units, *SMLI* Storz Medical Lithotripsy Index

Out of the cohort, 173 cases (30.9%) developed a radiographic perinephric hematoma on Day 2 imaging. Multivariate logistic regression identified that higher total energy delivered (odds ratio = 1.533, *p*-value = 0.003), a higher mean stone density (OR = 2.603, *p* = 0.01), a higher maximal stone density (OR = 3.578, *p* = 0.03), and lower pole stone status (OR = 1.545, *p* = 0.029) were risk factors of the development of perinephric hematoma. Adopting the stepwise ramping protocol (OR = 0.572, *p* = 0.042) was a protective factor against the development of hematoma (Table 2). Out of the patients that developed hematoma, 10 of them (1.7%) developed a symptomatic hematoma. The event rate was underpowered for further analysis for identifying the predicting factors of a symptomatic hematoma.

**Table 2:** Multivariate regression analysis for factors correlated with radiographic hematoma

Potential risk factors	Effect size	95% CI		*P* value
Maximum stone dimension	0.897	0.828	0.971	0.007
Mean stone density (divided by 1000)	2.603	1.253	5.406	0.01
Maximum stone density (divided by 1000)	3.578	1.135	11.28	0.03
Skin to stone distance	1.018	0.985	1.053	0.293
Mean cortical thickness	1.082	0.977	1.198	0.129
Lower pole stone status	1.545	1.047	2.281	0.029
Energy in SMLI (divided by 100)	1.005	1.002	1.008	0.003
Stepwise ramping protocol	0.572	0.288	0.856	0.042
Extended focal zone	1.131	0.716	1.788	0.596
Antiplatelet medication use	1.513	0.801	2.858	0.201
Age > 70	1.005	0.986	1.024	0.617
Hypertension	1.112	0.738	1.674	0.612

*SMLI* Storz Medical Lithotripsy Index

## Discussion and literature review

This analysis of two prospective trials reports that perinephric hematoma occurred in 30.9% of patients treated with primary SWL for renal calculi. Notably, none of the patients in our cohort presented with perinephric hematoma as an unplanned admission, which indicates that instances of symptomatic hematoma were not captured in this study. Our findings identified that risk factors for the development of radiographic perinephric hematoma include higher total energy delivered, higher stone density, absence of stepwise ramping protocol implementation, and lower pole stone status.

Renal hematoma is recognized as one of the most severe complications associated with SWL. Its effects vary widely, ranging from persistent loin pain that is not manageable with standard analgesics, to the development of renal abscess, and in rare cases, complications as severe as duodenal obstruction or hematoma with scrotal extension. While the reported incidence of perinephric hematoma in retrospective data is less than 1% [2, 7, 8], this rate increases significantly to 20–30% in prospective trials where post-procedural imaging such as CT or MRI is routinely employed [2, 9].

The mechanism involves shockwave-induced focal damage to the nephron and associated microvasculature, characterized by initial cellular fragmentation followed by secondary vacuolization and membrane bleeding [10, 11]. Histological examinations of human and animal models have shown immediate damage to glomerular and mid-sized arterial and venous structures following SWL [12, 13]. Additionally, renal tubules and surrounding vascular structures are susceptible to damage, which can result in microbleeding. This bleeding can lead to the accumulation of fluids and, subsequently, the formation of subcapsular or perirenal hematomas [14]. These hematomas can cause parenchymal fibrosis, leading to a loss of function, underscoring this complication's clinical significance [15, 16].

### Body mass index

Multiple potential risk factors have been implicated in the development of post-SWL hematomas. In a 418-patient matched case–control analysis, Nussberger et al. [17] reported a 9% incidence of perinephric hematoma following SWL. They discovered a high body mass index (BMI > 30) predicted perinephric hematoma. For patients with high BMI, the authors suggested that the longer skin-to-stone distance (SSD) due to subcutaneous adiposity affected the focal length and energy delivered by the lithotripter. In obese patients with concurrent metabolic syndrome, endothelial dysfunction or altered coagulation were postulated as contributing factors. It was also suggested that obesity, in conjunction with metabolic syndrome, creates a state of chronic inflammation, and increased oxidative stress could make the vascular endothelium and basement membrane more susceptible to shockwave damage [18, 19]. In the current study, we did not observe an effect of BMI on hematoma development in our cohort. As the majority of our cohort was Asian, the mean BMI was significantly lower than that of Caucasian cohorts, suggesting that the adverse effects of a very high BMI would be less likely to impact our patients.

### Energy delivered and stepwise ramping protocol

High-quality evidence suggests a renoprotective effect from adopting a stepwise ramping protocol. Skuginna et al. conducted a randomized trial involving 418 patients with either solitary or multiple renal stones. It was observed that a stepwise ramping regimen reduced the incidence of perinephric hematoma (detected via ultrasound) from 13% in the control group with a fixed voltage protocol to 5.6% in the ramping group [20]. A previous publication from our center, which was based on one of the cohorts adopted by the current analysis, also confirmed that ramping was a protective factor against the development of post-treatment hematoma [5], with a stepwise ramping protocol reducing instances of perinephric hematoma from 43.8% to 23.8% (*p* < 0.001). Shockwave-induced endothelial damage has been shown to have a dose-dependent relationship. In a stepwise ramping protocol, the overall energy delivered is reduced compared to a fixed voltage protocol. In fact, pretreating patients with low-energy shockwaves had been shown protect against SWL induced renal injury as early as in the 2000s [21–23]. Moreover, it has been suggested that low-voltage shocks delivered during ramping could induce vasoconstriction, making small and medium-sized vessels less susceptible to shockwave damage.

Interestingly, our investigation identified that lower polar status is a predictor of post-SWL hematoma formation. Unfortunately there is no published explainable mechanism nor established evidence that demonstrate the reasoning behind this observation. A postulation could include the potential higher energy used during SWL for lower pole stones, as a result of more difficult stone clearance due to an unfavorable calyceal anatomy [24, 25].

Overall, the collective findings of these trials indicate that a stepwise ramping protocol with lower total energy delivery can protect against perinephric hematoma without compromising stone fragmentation or the stone-free rate (from D2 stone-free rate that was reported by Ng et al. and 3-month stone-free rate that was reported by Skuginna et al.). Along with the conclusions of the current analysis, a stepwise ramping protocol should likely be employed in treating renal stones with SWL.

### Age, hypertension and cardiovascular disease

Hypertension has been suggested as a risk factor of post-SWL perinephric hematoma. Razvi et al. [26] analyzed 21 patients who developed perinephric hematoma in their retrospective cohort treated with SWL for proximal ureteric and renal stones. They reported that intraoperative hypertension, with systolic blood pressure (SBP) exceeding 140 (HR = 3.302), and the use of antiplatelet medication (HR = 4.198) were risk factors of post-SWL hematoma. It was postulated that vascular strain associated with hypertension makes the endothelium more susceptible to shockwave-induced damage [27]. Another retrospective cohort by Kostakopoulos [28], involving 23 cases of perinephric hematoma, also identified uncontrolled hypertension as a risk factor. Our study, however, did not reveal such a correlation, likely due to variations in how hypertension was defined across studies. In our research, hypertension was classified based on antihypertensives usage or if the patient was coded with hypertension according to ICD-9 criteria. In contrast, Razvi and colleagues based their findings on real-time blood pressure measurements during treatment.

A further retrospective study on 31 cases of perinephric hematoma following SWL [7] noted that hypertension and coagulation disorders are commonly reported in cases with hematoma, thus concluding these as potential risk factors for its development. The randomized controlled trial by Skuginna et al. [20] also identified advanced age as a risk factor for perinephric hematoma, reporting an odds ratio of 1.003 in univariate analysis. Similarly, Schnabel et al., from a cohort of 857 patients (reporting 7 cases of symptomatic hematoma detected by ultrasound) [29], concluded that older age and cardiovascular comorbidities are risk factors for perinephric hematoma. A 2004 study by Dhar et al. also indicated age as a risk factor, with an increased risk of 1.67-fold for each 10-year increment in patient age [9].

These factors likely have an intertwined relationship; with increased age comes greater vascular stiffness and fragility, thus rendering the blood vessel walls more susceptible to damage. The presence of medical comorbidities such as hypertension or cardiovascular disease further heightens the risk of renal hematoma in the elderly. However, as society ages, there will likely be an increasing number of elderly patients with stone diseases requiring treatment. The mere presence of hypertension or advanced age should not preclude them from receiving SWL as the primary treatment for stones. Careful patient selection is crucial, such as optimizing uncontrolled hypertension before proceeding with SWL.

### Antithrombotic medication

The evidence concerning the effects of antithrombotic medication on post-procedural hematoma remains inconclusive. Uncorrected coagulopathy is a recognized contraindication for shock wave lithotripsy. Only a limited number of studies have investigated the impact of such medications, given the clear risks involved. Zanetti et al. prospectively categorized 23 patients on antithrombotic medications according to their thromboembolic risk [30], with patients at low risk having their medication discontinued 8 days before the procedure, while those at high risk received bridging unfractionated heparin. No incidences of perinephric hematoma were observed. Conversely, a correlation between antiplatelet use and perinephric hematoma was noted in the cohort studied by Razvi et al. (HR = 4.198) [26]. In our current study, probably due to the cessation of medication for one week, no correlation was observed between its use and hematoma development. Ultimately, there is no robust prospective evidence suggesting patients using antiplatelet or blood-thinning medications are contraindicated for SWL, provided appropriate precautions are taken. Recommendations are based on expert consensus, thus necessitating a case-by-case evaluation of patients. The relevant literature that reported on the factors predicting the development of perinephric hematoma is summarized in Table 3.

**Table 3:** Summary of studies analyzing risk factors of post-SWL perinephric hematoma

Author	Year	Nature	Cases	Incidence of hematoma	Lithotripter used	Risk factors reported	Author’s comments/suggestions
Dhar et al. [9]	2004	Prospective registry	415	Radiograph: 17 (4.1%) Symptomatic: 3 (0.7%)	Storz Modulith SLX	Increasing age at treatment (OR 1.67 for each 10-year increment,* p* = 0.009); no significant association with mean arterial pressure	Subcapsular or perinephric hematoma is a frequent complication with SWL. Increasing patient age significantly raised the risk of hematoma
El-Nahas et al. [35]	2018	Retrospective	1179	3 (0.25%)	Dornier Lithotripter S	Solitary kidney (OR 2.855,* p* = 0.044), ASA II (OR 1.965,* p* = 0.007), hydronephrosis (RR 1.639,* p* = 0.024), stone length (RR 1.083, p < 0.001)	Hospital admission risks must be communicated to patients with identified risk factors. Elective management is conservative
Graber et al. [36]	2003	Prospective randomized trial	167	Not explicitly mentioned	HM3 (Dornier) and Lithostar Plus (Siemens)	Treatment with HM3 showed fewer complications and better stone disintegration compared to Lithostar Plus	The HM3 is recommended over Lithostar Plus due to better stone disintegration rates, fewer complications, and less post-treatment dilatation of the collecting system
Knapp et al. [37]	1988	Prospective registry	3,620 treatments	24 hematomas in 21 patients (0.66%)	HM3 Dornier lithotripter	Hypertension, particularly poorly controlled, pre-treatment urinary tract infection, and simultaneous bilateral treatment	Emphasized that hypertension is a significant risk factor for perinephric hematoma post-SWL. Recommended conservative management and careful monitoring of patients with risk factors
Kostakopoulos et al. [28]	1995	Retrospective	4,247	23 (0.54%)	Dornier HM-3 and HM-4 lithotripters	19 out of 23 patients with hematoma (82.6%) had hypertension; Slight increase in hematoma in over-50-year-old age group (0.62%)	Medical optimisation is needed prior to SWL treatment
Momose et al. [38]	1999	Prospective study	412 treatments on 397 patients	EDAP LT-01: 31 (16.6%) Siemens Lithostar: 44 (19.6%)	EDAP LT-01 and Siemens Lithostar	Subcapsular hematoma significantly higher with Siemens Lithostar (P < 0.0001)	The Siemens Lithostar exerts greater pressure on renal tissue, possibly leading to higher rates of subcapsular hematoma compared to the EDAP LT-01
Newman and Saltzman [39]	1991	Retrospective	1,012	6 clinically significant hematomas (0.59%)	Not Specified	Hypertension, diabetes mellitus, coronary artery disease, obesity	Identified hypertension, coronary artery disease, diabetes, and obesity as significant risk factors for developing clinically significant hematomas post-SWL
Ng et al. [5]	2019	Prospective randomized trial	300	Ramping group: 23.8% Fixed voltage group: 43.8%	Modulith SLX-F2	Ramping protocol significantly reduced the risk of perinephric hematoma compared to fixed voltage protocol (p < 0.001)	The study suggests that a ramping protocol in SWL not only maintains similar treatment success rates as fixed voltage protocols but also significantly reduces the incidence of perinephric hematomas
Ng et al. [6]	2022	Prospective randomized trial	320	Standard FZ group: 33.6%; Extended FZ group: 33.1%	Modulith SLX-F2	Focal zone is not shown to alter the rates of perinephric hematoma (* p* = 0.932)	N/A
Nussberger et al. [17]	2017	Prospective randomized trial	418	39 (9%)	Modulith SLX-F2	High (> 30) and low (< 21.5) BMI (Significant increase in risk, p < 0.001 for both)	Consider alternative endoscopic treatment options for patients with high or low BMI due to increased risk of renal damage. SWL effectiveness may be reduced and risk increased
Razvi et al. [26]	2012	Prospective registry	6172	21 (0.34%)	Storz Modulith SLX-F2	Intraoperative hypertension (HR 3.302,* p* = 0.0384), anticoagulant/antiplatelet drugs (HR 4.198,* p* = 0.0355)	Intraoperative hypertension and use of anticoagulant/antiplatelet drugs are significant risk factors
Schnabel et al. [29]	2014	Prospective study	1,324	7 (0.53%); 3 symptomatic, 4 asymptomatic	Siemens Lithoskop	Older age, vascular comorbidities; low-dose ASA not a risk factor	Highlighted the low incidence of hematoma (0.5%) with a smaller subset being symptomatic (0.23%). Emphasized conservative management and prospective studies for further examination
Serra et al. [7]	1999	Retrospective	10,953	31 (0.28%)	Siemens Lithostar	Hypertension (46% of cases), previous ESWL sessions, coagulation disorders	Renal hematoma post-SWL is rare but serious. Conservative management is generally sufficient. Hematoma is not a contraindication for further residual stone treatments
Skuginna et al. [20]	2015	Prospective randomized trial	418	Ramping group: 12 (5.6%) Fixed group: 205 (13%)	Modulith SLX-F2	Older age (OR 1.03, 95% CI 1.00–1.05,* p* = 0.04), Stepwise voltage ramping (OR 0.39, 95% CI 0.19–0.80,* p* = 0.01)	Stepwise voltage ramping during SWL was associated with a lower risk of renal damage without compromising treatment effectiveness

*OR* odds ratio, *BMI* body mass index, *95% CI* 95% confidence interval, *SWL* shock wave lithotripsy, *FZ* focal zone

### Type of lithotripter

In the current study, all of the procedures were performed by an electromagnetic lithotripter. A relatively dated comparative study demonstrated slightly higher rates of post-SWL hematoma in electromagnetic units compared to electrohydraulic units (3% vs 4%) whose difference, however, failed to reach a statistical significance [31]. Subsequent studies on electromagnetic lithotripter reported hematoma rate of ranging from less than 1% [32], to up to 13% [9, 33]. Meanwhile, the current study—with all patients receiving cross-sectional imaging regardless of symptoms—reported a rate of radiographic hematoma at 30%. As for piezoelectric lithotripter—one of the newer generation lithotripters with the ability a achieve a narrower focal zone and thus was postulated to improve SWL outcomes—a recent RCT in 2023 utilizing piezoelectric lithotripter with wide (8 mm) versus narrow (2 mm) focal zone reported a hematoma rate of 0–1.4% (34). However it was based on USG assessment only. Therefore one should take note that the reported rates of hematoma were highly affected by adequacy (imaging performed uniformly for the entire cohort versus performed only when patient presented with symptom), timing of imaging and modality of imaging (ultrasound versus cross-sectional imaging like CT or MRI). Unless we have updated head-to-head comparative cohorts between lithotripters with unified post-procedural imaging, it would be challenging to arrive at a conclusion as to whichever type of lithotripter is superior, in terms of post-SWL hematoma.

### Strengths and limitations

The merits of the current study must be acknowledged. Our cohort represents one of the larger groups studied prospectively in the contemporary era. It is known that the sensitivity of ultrasound for detecting perinephric hematoma is low. Using cross-sectional imaging for every case at a standardized timeframe after SWL provided a valid snapshot of perinephric hematoma secondary to shockwave lithotripsy. We believe this will be invaluable information when advising our patients on undergoing SWL for renal calculi, assisting our analysis in avoiding selection bias. Additionally, our cohort included well-defined information ranging from patients' baseline morbidities to treatment details and stone parameters. Therefore, the subsequent regression analysis of the risk factors of hematoma development likely offers a more comprehensive analysis than other available literature, which tends to be retrospective. However, it is important to mention the limitations of this study. Notably, a limited patients in our study suffered symptomatic hematoma. Therefore, we are unable to analyze whether there is a distinct set of risk factors for such morbidity.

## Conclusion

This study identifies key risk factors of perinephric hematoma following shockwave lithotripsy for renal calculi, offering insights into the procedural and stone-related factors that influence this serious complication. Our findings underscore the importance of contemporary treatment protocols, particularly the adoption of a stepwise ramping protocol, in mitigating the risks associated with SWL. They also shed light on the need for careful patient selection and pre-procedural optimization. Further research is still necessary to refine and validate these findings across diverse patient populations and clinical settings.

## Data Availability

The data could be made available upon request submitted to the corresponding author.
